# Epidermal growth factor receptor inhibition attenuates non-alcoholic fatty liver disease in diet-induced obese mice

**DOI:** 10.1371/journal.pone.0210828

**Published:** 2019-02-08

**Authors:** Sorim Choung, Ji Min Kim, Kyong Hye Joung, Eaum Seok Lee, Hyun Jin Kim, Bon Jeong Ku

**Affiliations:** 1 Department of Medical Science, College of Medicine, Chungnam National University, Daejeon, South Korea; 2 Department of Internal Medicine, College of Medicine, Chungnam National University School of Medicine, Daejeon, South Korea; University of Basque Country, SPAIN

## Abstract

Non-alcoholic fatty liver disease (NAFLD) is one of the main causes of chronic liver disease. NAFLD begins with excessive lipid accumulation in the liver and progresses to nonalcoholic steatohepatitis (NASH) and cirrhosis. NAFLD is closely linked to dysregulated hepatic lipid metabolism. Although recent studies have reported that epidermal growth factor receptor (EGFR) signaling regulates lipid metabolism, the roles of EGFR and EGFR inhibitors as modulators of lipid metabolism are largely unknown. Here, we investigated whether inhibiting EGFR using the EGFR tyrosine kinase inhibitor (TKI) PD153035 improves NAFLD. Our results demonstrate that EGFR was activated in liver tissues from high fat diet (HFD)-induced NAFLD mice. Inhibiting EGFR using PD153035 significantly reduced phosphatidylinositol-3-kinase/protein kinase B signaling and sterol responsive elementary binding protein 1 and 2 expression, which prevented HFD-induced hepatic steatosis and hypercholesterolemia by reducing de novo lipogenesis and cholesterol synthesis and enhancing fatty acid oxidation. Additionally, inhibiting EGFR improved HFD-induced glucose intolerance. In conclusion, these results indicate that EGFR plays an important role in NAFLD and is a potential therapeutic target.

## Introduction

The prevalence of non-alcoholic fatty liver disease (NAFLD) is increasing rapidly worldwide and it is now the most common liver disease, with an estimated global prevalence of ~25% [1]. NAFLD is characterized by excess fat accumulation in the liver, including simple fatty liver and nonalcoholic steatohepatitis (NASH). NAFLD can progress to liver cirrhosis and hepatocellular carcinoma [2], and is closely related to obesity and metabolic syndrome. Several previous studies reported that insulin resistance is also strongly associated with NAFLD and that NAFLD and type 2 diabetes mellitus frequently coexist [3,4]. Although NAFLD has become a public health concern worldwide, there is still no approved drug for the treatment of NAFLD.

The epidermal growth factor receptor (EGFR) signaling pathway has been implicated in many human diseases, especially in cancer, as it plays a central role in regulating the survival, proliferation, migration, and differentiation of various tissues [5]. Several studies have also suggested that EGFR is associated with metabolic disorders [6,7]. Our previous study showed that serum cholesterol and intrahepatic lipid levels were increased in mice with conditional ablation of mitogen-inducible gene 6 (Mig-6), an EGFR negative feedback inhibitor, in the liver [8], suggesting that EGFR is a possible target for the treatment of dyslipidemia and NAFLD. However, the effectiveness of EGFR-targeted treatments in NAFLD is unknown. Therefore, we aimed to investigate whether inhibiting EGFR using the EGFR tyrosine kinase inhibitor (TKI) PD153035 improves NAFLD.

## Materials and methods

### Animals

Male C57BL/6J mice were purchased from Harlan (Indianapolis, IN, USA). A high-fat diet (HFD) composed of 60% fat was purchased from Research Diets Inc. (D12492; New Brunswick, NJ, USA). The animals were maintained in a controlled environment (12 h light/12 h dark cycle; 50–60% humidity; ambient temperature 22 ± 2°C). Eight-week-old male mice were fed a normal chow diet (NCD) or HFD for 8 consecutive weeks and then divided randomly into three groups: the NCD group were fed an NCD without treatment, the HFD group were fed a HFD without treatment, and the HFD+PD group were fed a HFD and treated with PD153035 (30 mg/kg/day O.G.; Selleck Chemicals, Houston, TX, USA) for the final 4 weeks. All animals received humane care according to institutional guidelines, and all experimental procedures were approved by the Institutional Review Board of Chungnam National University School of Medicine (Daejeon, South Korea).

### Cell culture

The Huh-7 hepatocellular carcinoma cell line was purchased from the American Type Culture Collection (Manassas, VA, USA) and cultured according to the manufacturer’s instructions. Cells were cultured in high-glucose Dulbecco’s Modified Eagle’s Medium (DMEM; Invitrogen, Carlsbad, CA, USA) supplemented with 10% fetal bovine serum (FBS; Invitrogen) and 1% penicillin-streptomycin. Huh-7 cells were stimulated with 10 ng/mL EGF (Sigma, St. Louis, MO, USA) and treated with 10 μM gefitinib (Sigma). Huh-7 cells were incubated with 400 μM palmitic acid and 10 μM gefitinib for 24 h.

### Histological analysis

Tissue samples were obtained from 18-week-old mice. Samples for light microscopy were fixed in 4% paraformaldehyde (PFA) for 1 h. Paraffin embedding, sectioning, and hematoxylin and eosin (H&E) and oil red O staining were performed according to standard protocols.

### Serum biochemical measurements

Blood was collected from the heart under general anesthesia. Samples were centrifuged at 5,000 rpm for 5 min and the supernatants were collected. Serum insulin levels were measured by enzyme-linked immunosorbent assay (ELISA; ALPCO Diagnostics, Salem, NH, USA) using kits obtained from the indicated supplier. Biochemical analyses, including measuring free fatty acid and total cholesterol levels, were performed using a Hitachi 7180 auto analyzer and Wako reagents (Wako Pure Chemical Industries, Ltd., Osaka, Japan).

### Western blotting

Livers from mice and Huh-7 cells were lysed in RIPA buffer (30 mmol/L Tris, pH 7.5, 150 mmol/L sodium chloride, 1  mmol/L phenylmethylsulfonyl fluoride, 1 mmol/L sodium orthovanadate, 1% Nonidet P-40, 10% glycerol, and phosphatase and protease inhibitors). Western blotting was performed on 30–50-μg protein samples using commercially available antibodies against the following antigens: EGFR, phospho-EGFR (pEGFR), Akt, phospho-Akt (pAkt), fatty acid synthase (FASN), anti-sterol responsive elementary binding protein 1 (SREBP1), and SREBP2 (all from BD Biosciences, San Jose, CA, USA). Secondary antibodies (goat anti-mouse and goat anti-rabbit) were obtained from Cell Signaling Technology, Inc. (Beverly, MA, USA).

### RNA isolation and real-time PCR

Total RNA was isolated using TRIzol reagent (Thermo Fisher Scientific, Waltham, MA, USA) and cDNA was prepared from total RNA using M-MLV reverse transcription and oligo-dT primers (Invitrogen). The resulting cDNA was amplified on a Rotor-Gene 6000 real-time rotary analyzer with software version 1.7 (Corbett Life Science, Sydney, Australia). Real-time polymerase chain reaction (PCR) was performed using cDNA and QuantiTect SYBRGreen PCR Master Mix (Qiagen, Hilden, Germany). All quantitative calculations were performed using the ΔΔCT method. The comparative Ct method was used to quantify transcripts, which were normalized to 18sRNA expression. Values were expressed as fold changes vs. control groups.

### Statistical analysis

Statistical analyses were performed using Stat Graph Prism 5 (GraphPad Software, Inc., La Jolla, CA, USA). Data are presented as the mean ± standard error of the mean (SEM). To compare values obtained from two or more groups, data from animal studies were analyzed using two-way repeated-measures analysis of variance followed by Bonferroni’s correction for multiple comparisons, one-way analysis of variance followed by Tukey’s post hoc test, or two-tailed Student’s *t-*tests. *P* < 0.05 was considered statistically significant.

## Results

### Inhibiting EGFR reverses HFD-induced weight gain

**Fig 1:**
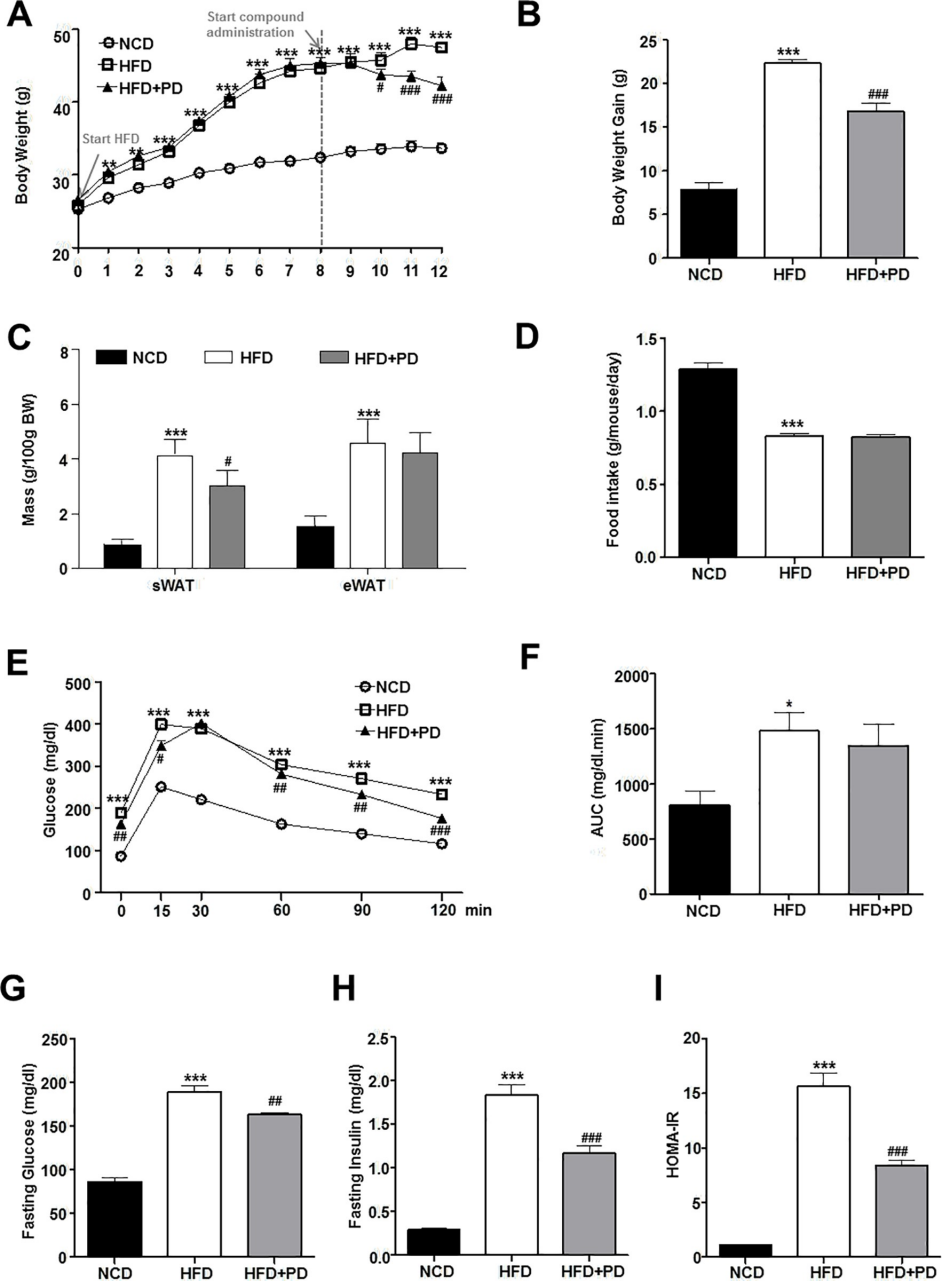
Effect of epidermal growth factor receptor (EGFR) inhibition on metabolic phenotypes in high fat diet (HFD)-fed mice. (A) Body weight. (B) Body weight gain. (C) Subcutaneous and epididymal fat mass (g/100 g body weight). (D) Food intake (g/day/mouse). (E) Glucose tolerance test (GTT); glucose (1 g/kg body weight) was injected into overnight-fasted mice. (F) Area under the curve (AUC) for GTT. (G) Fasting blood glucose levels. (H) Fasting blood insulin levels. (I) Homeostasis model of assessment for insulin resistance index (HOMA-IR) assessed as fasting insulin (μU/mL) × fasting glucose (mg/dL)/22.5. NCD, normal chow diet (NCD)-fed mice (NCD, black bars or white circles); HFD, untreated HFD-fed mice (white bars or white squares); HFD+PD, HFD-fed mice treated with PD153035 (gray bars or black triangles). Data are expressed as the mean ± standard error of the mean (SEM). * *P* < 0.05, ** *P* < 0.001, *** *P* < 0.0001 NCD vs. HFD group. # *P* < 0.05, ## *P* < 0.001, ### *P* < 0.0001 HFD vs. HFD+PD group.

Male C57BL/6J mice were maintained on an NCD or HFD for 8 weeks. A significant increase in body weight was observed in the HFD group compared with the NCD group, as expected (Fig 1A and 1B). At the end of week 8, HFD-fed mice were treated with the specific EGFR inhibitor PD153035. PD153035 is a highly potent and specific inhibitor of EGFR tyrosine kinase; it suppresses the tyrosine phosphorylation and activation of EGFR by competitively binding to the receptor tyrosine kinase domain [9]. Treatment with PD153035 reversed HFD-induced weight gain throughout the 4-week treatment period (Fig 1A and 1B). In addition, the weights of subcutaneous and epididymal fat were reduced significantly in the HFD+PD group compared with the HFD group (Fig 1C). During the 4-week treatment period, daily food intake was similar between the HFD and HFD+PD groups (data not shown).

### Inhibiting EGFR improves glucose tolerance and insulin sensitivity in HFD-induced obese mice

To compare glucose homeostasis parameters among groups, glucose tolerance tests were performed. Treatment with PD153035 for 4 weeks improved glucose tolerance (Fig 1D) and reduced fasting plasma glucose levels (Fig 1E) in HFD-induced obese mice. In addition, plasma insulin levels were significantly lower in the HFD+PD group compared with the HFD group (Fig 1F). Treatment with PD153035 also improved the homeostasis model of assessment for insulin resistance index (HOMA-IR) (Fig 1G). These findings indicate that EGFR inhibition reduces HFD-induced weight gain, leading to improved glucose tolerance and insulin sensitivity.

### Inhibiting EGFR reduces serum lipid levels in HFD-induced obese mice

**Fig 2:**
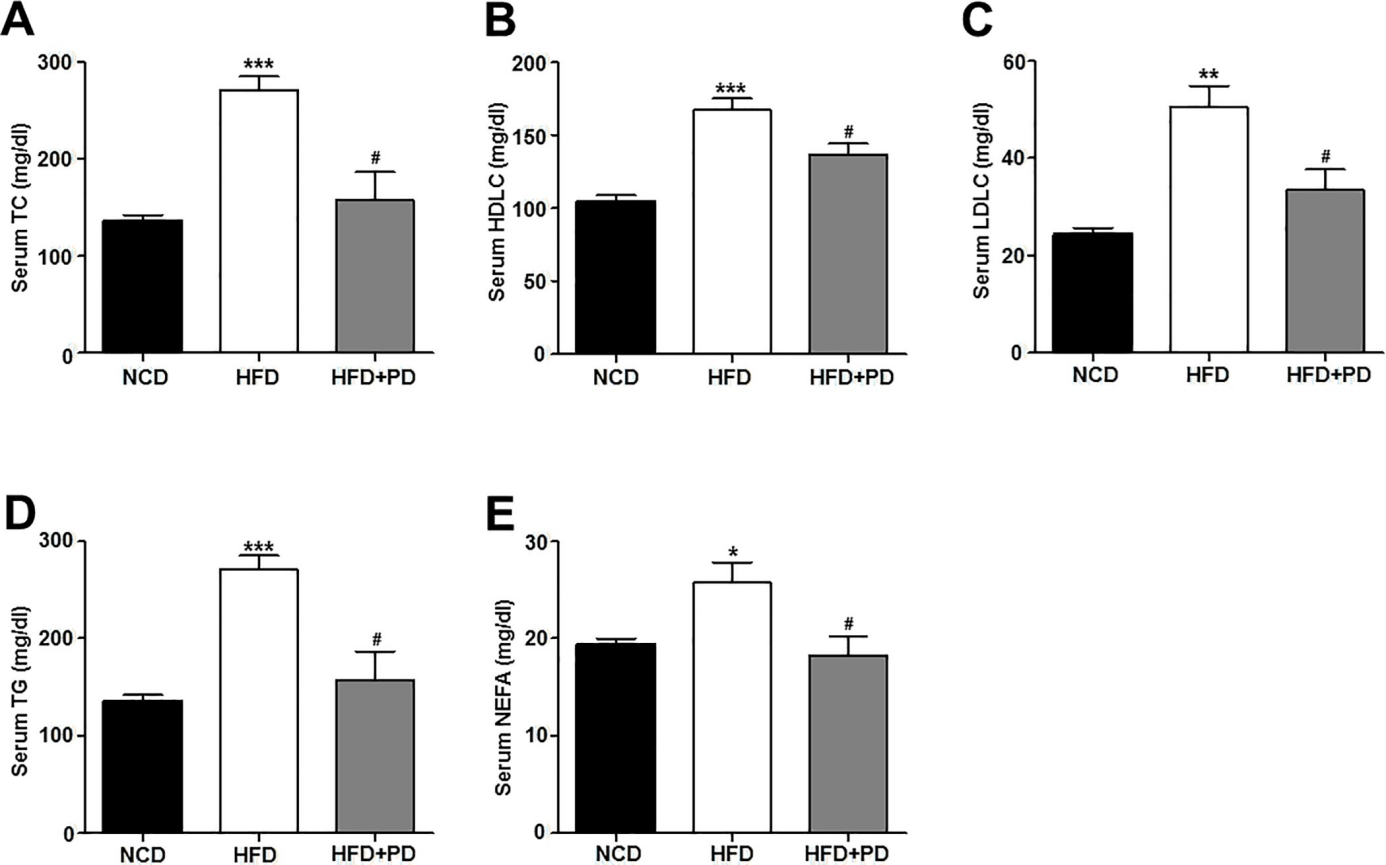
Effects of EGFR inhibition on serum lipid levels in HFD-fed mice. Serum concentrations were measured after 12 weeks of NCD, HFD, and HFD+PD treamtment. (A) Serum total cholesterol (TC) concentration (mg/dL). (B) HDL cholesterol concentration (mg/dL). (C) LDL cholesterol concentration (mg/dL). (D) Triglyceride (TG) concentration (mg/dL). (E) Non-esterified fatty acid (NEFA) levels (mg/dL). NCD, NCD-fed mice (NCD, black bars); HFD, untreated HFD-fed mice (white bars); HFD+PD, HFD-fed mice treated with PD153035 (gray bars). Data are expressed as the mean ± SEM. * *P* < 0.05, ** *P* < 0.001, *** *P* < 0.0001 NCD vs. HFD group. # *P* < 0.05, ## *P* < 0.001, ### *P* < 0.0001 HFD vs. HFD+PD group.

Because NAFLD was strongly linked with dyslipidemia, we next investigate serum lipid levels in each group. HFD-fed mice exhibited significantly increased serum total cholesterol (TC), high density lipoprotein (HDL) cholesterol, low density lipoprotein (LDL) cholesterol, triglyceride (TG), and non-esterified fatty acid (NEFA) levels compared with those of the NCD group (Fig 2A–2E). Treatment with PD153035 significantly reduced serum TC, HDLC, and LDLC levels in HFD-fed mice to levels close to those observed in NCD-fed mice (Fig 2A–2C). Serum TG and NEFA levels were also significantly lower in the HFD+PD group than in the HFD group (Fig 2D and 2E).

### Inhibiting EGFR prevents hepatic steatosis in HFD-induced obese mice

**Fig 3:**
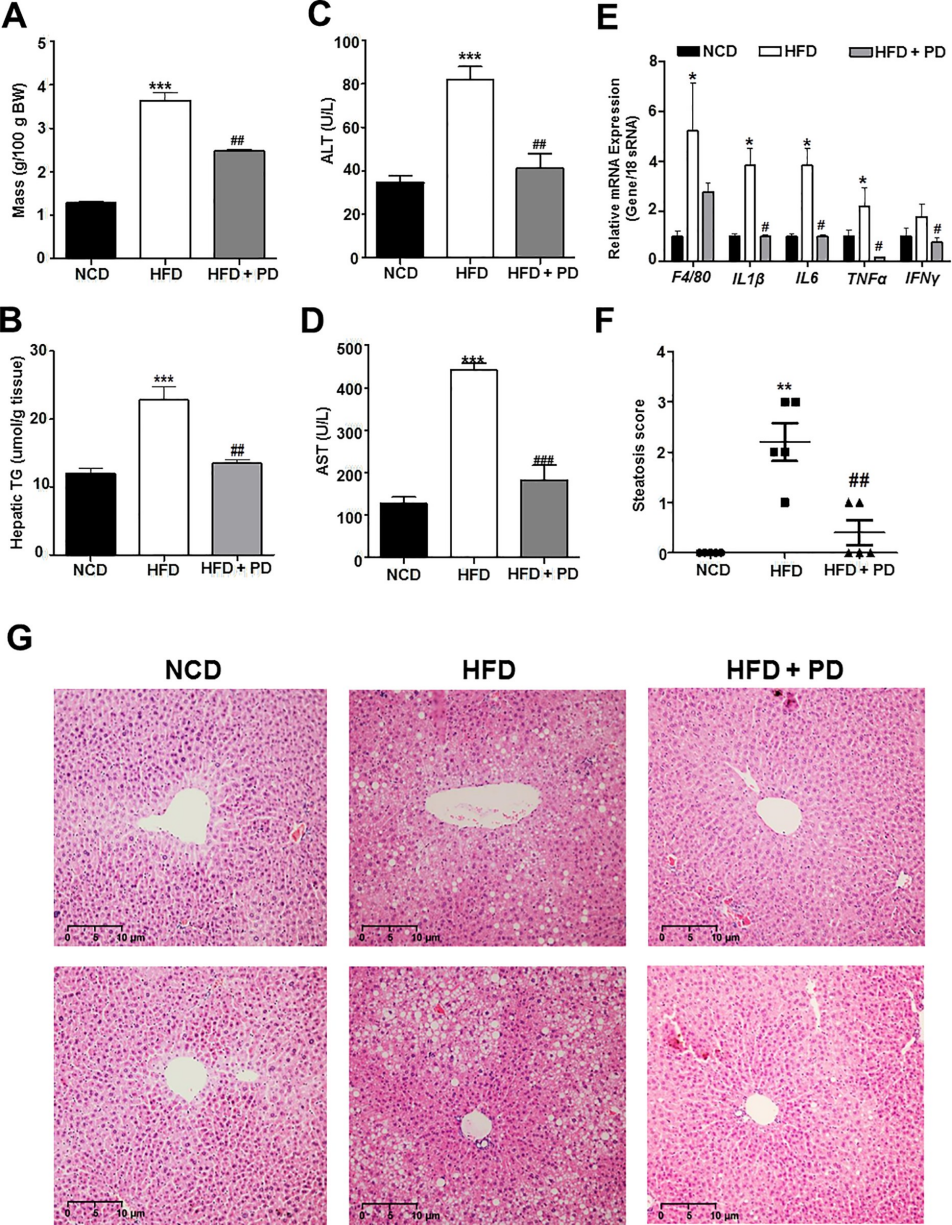
Effect of EGFR inhibition on hepatic steatosis in HFD-fed mice. (A) The weight of liver tissue (g/100 g body weight). (B) Hepatic triglyceride(TG) level (μmol/g tissue). (C) Alanine transaminase (ALT) (U/L) levels. (D) Aspartate transaminase (AST) (U/L) levels. (E) The m RNA levels of selected inflammatory genes were showed by real time PCR for liver tissue. (F) Steatosis score. Pathology scores were as follows: 0, no significant lesions (0%); 1, minimal (<10%); 2, mild (10–25%); 3, moderate (25–40%); 4, marked (40–50%); and 5, severe (>50%) (G) Representative images of H&E staining of liver sections. Scale bars = 10 μm. NCD, NCD-fed mice (NCD, black bars); HFD, untreated HFD-fed mice (white bars); HFD+PD, HFD-fed mice treated with PD153035 (gray bars). Data are expressed as the mean ± SEM. * *P* < 0.05, ** *P* < 0.001, *** *P* < 0.0001 NCD vs. HFD group. # *P* < 0.05, ## *P* < 0.001, ### *P* < 0.0001 HFD vs. HFD+PD group.

The most common cause of hepatomegaly is hepatic steatosis, which is also the hallmark feature of NAFLD [10]. Furthermore, NAFLD is now the most common cause of elevated liver enzymes, indicating liver damage [11]. Therefore, we next compared liver weights and liver function tests, including measuring serum aspartate transaminase (AST) and alanine transaminase (ALT) levels, between groups. HFD-fed mice had a higher mean liver weight and elevated liver enzymes compared with NCD-fed mice (Fig 3A–3C). As expected, treatment with PD153035 significantly reduced liver weight (Fig 3A) in HFD-fed mice. Treatment with PD153035 also significantly reduced AST and ALT levels in HFD-fed mice (Fig 3B and 3C).

To evaluate the intrahepatic lipid contents and the degree of liver damage, we performed H&E staining of liver tissues. Histopathological analysis of the livers in the HFD group revealed markedly increased hepatic steatosis (Fig 3D), whereas treatment with PD153035 noticeably reduced hepatic lipid contents. Hepatocellular vacuolation, which indicates hepatocellular damage, was also reduced by treatment with PD153035 (Fig 3D). These results suggest that inhibiting EGFR may improve HFD-induced hepatic steatosis.

### Inhibiting EGFR regulates hepatic lipid metabolism in HFD-induced obese mice

**Fig 4:**
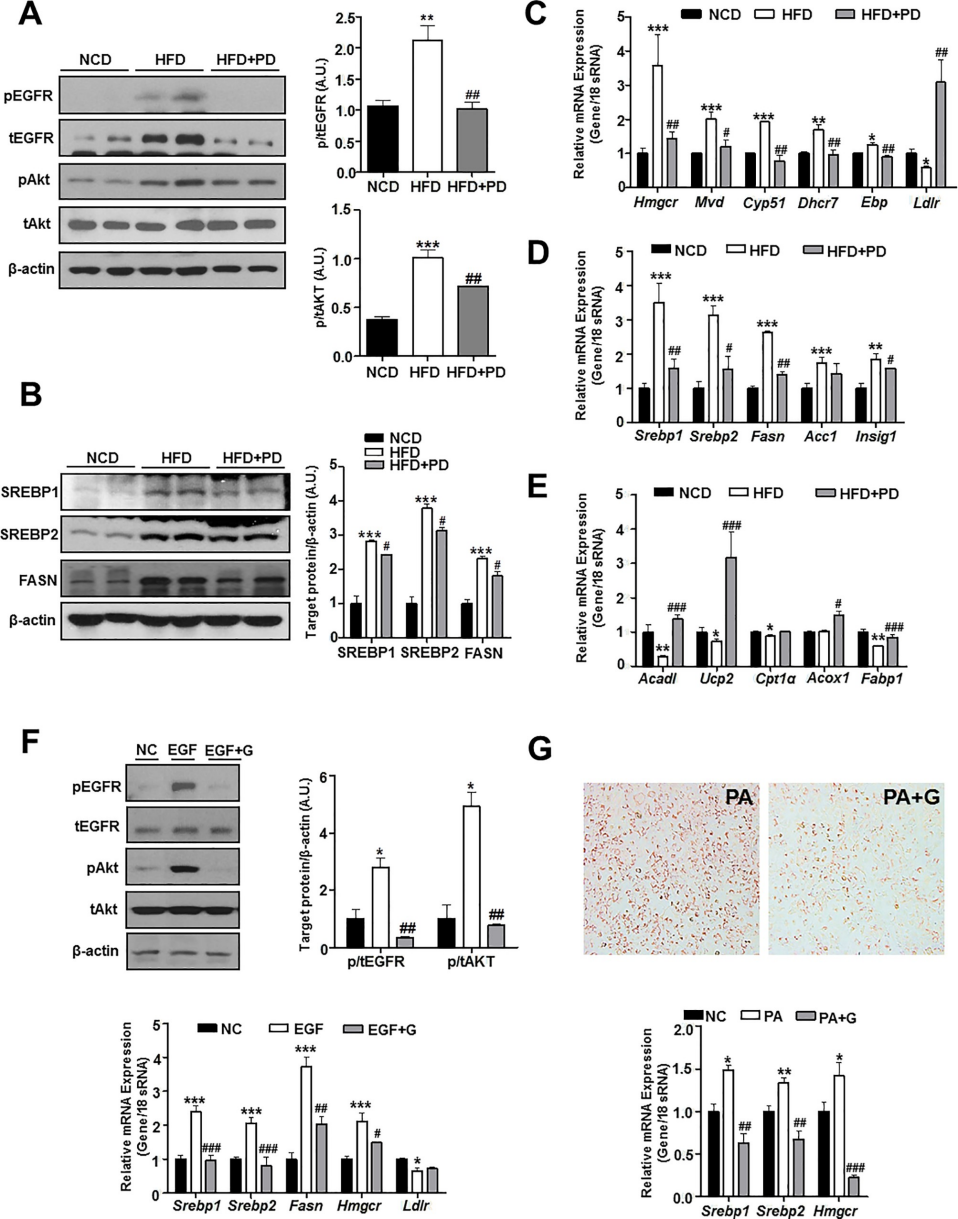
Effects of EGFR inhibition on hepatic lipid metabolism in HFD-fed mice. (A) Analysis of proteins in the EGFR signaling pathway in mouse liver tissues. The figure shows the total and phosphorylated forms of EGFR and Akt. (B) Analysis of proteins associated with de novo lipogenesis in mouse liver tissues. (C–E) Real time quantitative PCR showing the transcriptional levels of genes related to lipid and cholesterol metabolism. (C) Transcriptional levels of cholesterol biosynthesis-related genes in liver tissues. (D) Transcriptional levels of de novo lipogenesis-related genes in liver tissues. (E) Transcriptional levels of fatty acid β-oxidation-related genes in liver tissues. (F) Huh-7 cells were treated with EGF and gefitinib. The expression of proteins in the EGFR signaling pathway were measured in Huh-7 cells. Real-time quantitative PCR assay showing the transcriptional levels of lipid metabolism-related genes. (G) Huh-7 cells were treated with palmitic acid (PA) and gefitinib. Representative images of oil Red O staining of Huh-7 cells and the transcriptional levels of lipid metabolism-related genes, as examined by real-time quantitative PCR. NCD, NCD-fed mice (NCD, black bars); HFD, untreated HFD-fed mice (white bars); HFD+PD, HFD-fed mice treated with PD153035 (gray bars); NC, negative control; EGF, Huh-7 cells stimulated with 10 ng/mL EGF; EGF+G, Huh-7 cells stimulated with 10 ng/mL EGF and 10 μM gefitinib; PA, Huh-7 cells incubated with 400 μM palmitic acid; PA+G, Huh-7 cells incubated with 400 μM palmitic acid and 10 μM gefitinib for 24 h. Data are expressed as the mean ± SEM. * *P* < 0.05, ** *P* < 0.001, *** *P* < 0.0001 NCD vs. HFD group. # *P* < 0.05, ## *P* < 0.001, ### *P* < 0.0001 HFD vs. HFD+PD group.

To understand the role of EGFR in regulating hepatic lipid metabolism, we investigated whether inhibiting EGFR alters the mRNA and protein expression of proteins associated with hepatic lipid metabolism pathways such as hepatic de novo lipogenesis (DNL) and cholesterol synthesis. First, we examined whether EGFR signaling is activated in the livers of HFD-fed mice. The phosphorylation of EGFR and its downstream protein Akt was increased in the livers of HFD-fed mice. Treatment with PD153035 effectively reduced the phosphorylation of both proteins (Fig 4A).

We then examined the expression of sterol regulatory element-binding protein-1 (SREBP-1), a critical transcription factor for lipogenesis [12]. Abnormally increased hepatic DNL levels are closely linked to the development of NAFDL [13]. The protein expression of SREBP-1 and its lipogenic targets such as fatty acid synthase (FASN) was increased in HFD-induced NAFLD, whereas inhibiting EGFR decreased SREBP-1 and FASN expression. The expression of SREBP-2, a transcription factor that preferentially activates genes involved in cholesterol synthesis, was also decreased after EGFR inhibition (Fig 4B). These findings indicate that inhibiting EGFR reduces protein expression related to lipogenesis and cholesterol synthesis.

We further investigated the transcriptional levels of genes related to lipid and cholesterol metabolism. The mRNA levels of key genes for cholesterol biosynthesis, such as *HMG-CoA reductase (Hmgcr)*, *mevalonate diphospho decarboxylase (Mvd)*, *cytochrome P450 family 511 (Cyp51)*, *7-dehydrocholesterol reductase (Dhcr7)*, *and cholestenol delta-isomerase (Ebp)* were remarkably decreased by treatment with PD153035 (Fig 4C). In contrast, the mRNA expression of *low density lipoprotein receptor (Ldlr)* was increased following treatment with PD153035 (Fig 4C). The transcriptional levels of DNL-related genes, such as *Srebp1*, *Srebp2*, *Fasn*, *acetyl-CoA carboxylase 1* (*Acc1)*, *and insulin induced gene 1 (Insig1)*, were markedly decreased by treatment with PD153035 (Fig 4D). In addition, the mRNA levels of fatty acid β-oxidation-associated genes such as *acyl-CoA dehydrogenase-1 (Acad1)*, *uncoupling protein-2 (Ucp2)*, *carnitine palmitoyltransferase-1α (Cpt1α)*, *acyl-Coenzyme A oxidase-1 (Acox1)*, and *fatty acid binding protein-1 (Fabp1)* were decreased by treatment with PD153035 (Fig 4E). These changes ultimately led to reduced lipid accumulation in the liver.

### Inhibiting EGFR prevents intracellular lipid accumulation in hepatocytes

To reconfirm our results *in vitro*, we used the human hepatocellular carcinoma cell line, Huh-7. Cells were treated with EGF to stimulate the EGFR signaling pathway, and the activation was reversed by the EGFR TKI, gefitinib (Fig 4F). Consistent with the *in vivo* data, EGFR inhibition decreased the transcriptional level of genes involved in DNL and cholesterol biosynthesis, such as *Srebp1*, *Srebp2*, *and Hmgcr* (Fig 4F). Then, Huh-7 cells were treated with palmitic acid to simulate excessive free fatty acid influx into hepatocytes [14]. After 24 h, cells were stained with oil red O to examine intracellular lipid accumulation. Significantly increased intracellular lipid accumulation was observed in cells exposed to palmitic acid. However, inhibiting EGFR using gefitinib attenuated palmitic acid-induced lipid accumulation and DNL (Fig 4G).

## Discussion

The mechanisms of NAFLD progression have been studied in recent years. Although several mechanisms have been revealed [15,16], the pathogenesis of NAFDL is not fully understood. In this study, we confirmed that EGFR signaling is associated with the development of NAFLD. Our results demonstrated that EGFR signaling is increased in HFD-induced NAFLD. Moreover, EGFR inhibition using PD153035 induced a significant improvement in hepatic steatosis, which was accompanied by improved intrahepatic lipid levels. In addition, PD153035 also prevented HFD-induced dyslipidemia and insulin resistance. These results suggest that inhibiting the EGFR signaling pathway may be a potential therapeutic target for NAFLD and related dyslipidemia and insulin resistance.

NAFLD is classified into four stages as follows: stage 1, simple fatty liver (steatosis); stage 2, non-alcoholic steatohepatitis (NASH); stage 3, fibrosis; and stage 4, cirrhosis. In the early stages of NAFLD, only hepatic TG accumulation, without abnormal liver function, is found. NASH is characterized by hepatic steatosis with inflammation, and persistent inflammation can ultimately lead to irreversible fibrotic change [17]. Therefore, early diagnosis and intervention for hepatic steatosis are essential to prevent progression to fibrosis. According to the current results, EGFR signaling may be a novel target for the treatment of hepatic steatosis and steatohepatitis, which are the early stages of NAFLD.

EGFR, also known as ErbB1/HER1, is a member of the ErbB family of receptor tyrosine kinases. EGFR dimerization is a crucial initial step in EGFR signaling. EGFR dimerization stimulates the auto-phosphorylation of tyrosine residues, leading to the activation of several downstream pathways. Three major downstream signaling pathways activated by EGFR have been identified: (1) the rat sarcoma (RAS)/rapidly accelerated fibrosarcoma (RAF)/mitogen-activated protein kinase (MAPK) pathway; (2) the phosphatidylinositol-3-kinase (PI3K)/protein kinase B (Akt) pathway; and (3) the Janus kinase (JAK)/signal transducers and activators of transcription (STAT) pathway [18,19]. EGFR signaling is involved in cell growth and is frequently upregulated in various cancers such as lung, colon, and brain cancers [5,20]. Many studies have investigated the role of EGFR in cancer metabolism, particularly glucose metabolism in cancer cells [6,21]. For example, EGFR signaling promotes not only glycolysis, but also de novo fatty acid synthesis via the PI3K/Akt signaling pathway in cancer cells [22]. PI3K/Akt signaling upregulates SREBP-1, a master transcriptional regulator of fatty acid biosynthesis, and eventually promotes DNL and decreases fatty acid β-oxidation. PI3K/Akt signaling also upregulates SREBP-2, which is responsible for activating genes involved in cholesterol biosynthesis [23–26]. Previous reports have suggested that multiple factors, such as increased DNL and fatty acid uptake, reduced fatty acid oxidation, and decreased very low-density lipoprotein (VLDL) secretion, cause excess lipid accumulation in the liver [27]. The current study confirmed that PD153035 significantly reduced PI3K/Akt signaling and SREBP-1 and 2 expression, preventing HFD-induced hepatic steatosis by reducing DNL and cholesterol synthesis and enhancing fatty acid oxidation.

The current study also confirmed that inhibiting EGFR improves glucose tolerance. Several studies have suggested that EGFR affects glycolysis and glucose transport through the downstream RAS/RAF/MAPK and PI3K/Akt pathways [21]. In addition, previous reports indicated that EGFR TKIs may exert antidiabetic effects in type 1 and type 2 diabetes [28]. Although several mechanisms have been proposed to explain how TKIs can regulate glucose metabolism, the underlying mechanism is not fully understood. However, reduced insulin resistance, caused by reducing the expression of tumor necrosis factor (TNF)-α and interleukin (IL)-6 and inhibited M1 pro-inflammatory macrophage infiltration in adipose tissue, was suggested as a potential mechanism [29]. Because insulin resistance is closely associated with NAFLD, reducing cytokine levels and M1 macrophage infiltration may be additive mechanisms through which EGFR inhibition improves NAFLD. However, we did not examine the expression of TNF-α and IL-6 or the degree of macrophage infiltration, which is a limitation of our study.

## Conclusions

In conclusion, we demonstrated that inhibiting EGFR significantly improved HFD-induced NAFLD. To our knowledge, this is the first time that the role of EGFR as a therapeutic target of NAFLD has been experimentally investigated. Furthermore, we confirmed that inhibiting EGFR reduces liver fat accumulation and hepatocellular damage by reducing DNL and cholesterol synthesis and by enhancing fatty acid oxidation. However, further studies are needed to address the clinical efficacy and safety of EGFR TKIs such as PD153035.

## Supporting information

S1 Table Dataset. (XLSX)

## References

[1] Younossi ZM, Koenig AB, Abdelatif D, Fazel Y, Henry L, et al (2016) Global epidemiology of nonalcoholic fatty liver disease-Meta-analytic assessment of prevalence, incidence, and outcomes. Hepatology 64: 73–84. 10.1002/hep.28431 26707365

[2] Farrell GC, Larter CZ (2006) Nonalcoholic fatty liver disease: from steatosis to cirrhosis. Hepatology 43: S99–s112. 10.1002/hep.20973 16447287

[3] Adams LA, Waters OR, Knuiman MW, Elliott RR, Olynyk JK (2009) NAFLD as a risk factor for the development of diabetes and the metabolic syndrome: an eleven-year follow-up study. Am J Gastroenterol 104: 861–867. 10.1038/ajg.2009.67 19293782

[4] Milic S, Lulic D, Stimac D (2014) Non-alcoholic fatty liver disease and obesity: biochemical, metabolic and clinical presentations. World J Gastroenterol 20: 9330–9337. 10.3748/wjg.v20.i28.9330 25071327 PMC4110564

[5] Seshacharyulu P, Ponnusamy MP, Haridas D, Jain M, Ganti AK, et al (2012) Targeting the EGFR signaling pathway in cancer therapy. Expert Opin Ther Targets 16: 15–31. 10.1517/14728222.2011.648617 22239438 PMC3291787

[6] Makinoshima H, Takita M, Matsumoto S, Yagishita A, Owada S, et al (2014) Epidermal growth factor receptor (EGFR) signaling regulates global metabolic pathways in EGFR-mutated lung adenocarcinoma. J Biol Chem 289: 20813–20823. 10.1074/jbc.M114.575464 24928511 PMC4110289

[7] Bian Y, Yu Y, Wang S, Li L (2015) Up-regulation of fatty acid synthase induced by EGFR/ERK activation promotes tumor growth in pancreatic cancer. Biochem Biophys Res Commun 463: 612–617. 10.1016/j.bbrc.2015.05.108 26043686

[8] Ku BJ, Kim TH, Lee JH, Buras ED, White LD, et al (2012) Mig-6 plays a critical role in the regulation of cholesterol homeostasis and bile acid synthesis. PLoS One 7: e42915 10.1371/journal.pone.0042915 22912762 PMC3422237

[9] Yewale C, Baradia D, Vhora I, Patil S, Misra A (2013) Epidermal growth factor receptor targeting in cancer: a review of trends and strategies. Biomaterials 34: 8690–8707. 10.1016/j.biomaterials.2013.07.100 23953842

[10] Reid AE (2001) Nonalcoholic steatohepatitis. Gastroenterology 121: 710–723. 11522755 10.1053/gast.2001.27126

[11] Ekstedt M, Franzen LE, Mathiesen UL, Thorelius L, Holmqvist M, et al (2006) Long-term follow-up of patients with NAFLD and elevated liver enzymes. Hepatology 44: 865–873. 10.1002/hep.21327 17006923

[12] Eberle D, Hegarty B, Bossard P, Ferre P, Foufelle F (2004) SREBP transcription factors: master regulators of lipid homeostasis. Biochimie 86: 839–848. 10.1016/j.biochi.2004.09.018 15589694

[13] Fabbrini E, Sullivan S, Klein S (2010) Obesity and nonalcoholic fatty liver disease: biochemical, metabolic, and clinical implications. Hepatology 51: 679–689. 10.1002/hep.23280 20041406 PMC3575093

[14] Joshi-Barve S, Barve SS, Amancherla K, Gobejishvili L, Hill D, et al (2007) Palmitic acid induces production of proinflammatory cytokine interleukin-8 from hepatocytes. Hepatology 46: 823–830. 10.1002/hep.21752 17680645

[15] Malaguarnera M, Di Rosa M, Nicoletti F, Malaguarnera L (2009) Molecular mechanisms involved in NAFLD progression. J Mol Med (Berl) 87: 679–695. 19352614 10.1007/s00109-009-0464-1

[16] Marra F, Gastaldelli A, Svegliati Baroni G, Tell G, Tiribelli C (2008) Molecular basis and mechanisms of progression of non-alcoholic steatohepatitis. Trends Mol Med 14: 72–81. 10.1016/j.molmed.2007.12.003 18218340

[17] Serfaty L, Lemoine M (2008) Definition and natural history of metabolic steatosis: clinical aspects of NAFLD, NASH and cirrhosis. Diabetes Metab 34: 634–637. 10.1016/S1262-3636(08)74597-X 19195623

[18] Normanno N, De Luca A, Bianco C, Strizzi L, Mancino M, et al (2006) Epidermal growth factor receptor (EGFR) signaling in cancer. Gene 366: 2–16. 10.1016/j.gene.2005.10.018 16377102

[19] Huang L, Fu L (2015) Mechanisms of resistance to EGFR tyrosine kinase inhibitors. Acta Pharm Sin B 5: 390–401. 10.1016/j.apsb.2015.07.001 26579470 PMC4629442

[20] Sordella R, Bell DW, Haber DA, Settleman J (2004) Gefitinib-sensitizing EGFR mutations in lung cancer activate anti-apoptotic pathways. Science 305: 1163–1167. 10.1126/science.1101637 15284455

[21] Ru P, Williams TM, Chakravarti A, Guo D (2013) Tumor metabolism of malignant gliomas. Cancers (Basel) 5: 1469–1484. 24217114 10.3390/cancers5041469 PMC3875949

[22] Yamauchi Y, Furukawa K, Hamamura K, Furukawa K (2011) Positive feedback loop between PI3K-Akt-mTORC1 signaling and the lipogenic pathway boosts Akt signaling: induction of the lipogenic pathway by a melanoma antigen. Cancer Res 71: 4989–4997. 10.1158/0008-5472.CAN-10-4108 21632551

[23] Horton JD, Goldstein JL, Brown MS (2002) SREBPs: activators of the complete program of cholesterol and fatty acid synthesis in the liver. J Clin Invest 109: 1125–1131. 10.1172/JCI15593 11994399 PMC150968

[24] Krycer JR, Sharpe LJ, Luu W, Brown AJ (2010) The Akt-SREBP nexus: cell signaling meets lipid metabolism. Trends Endocrinol Metab 21: 268–276. 10.1016/j.tem.2010.01.001 20117946

[25] Jeon TI, Osborne TF (2012) SREBPs: metabolic integrators in physiology and metabolism. Trends Endocrinol Metab 23: 65–72. 10.1016/j.tem.2011.10.004 22154484 PMC3273665

[26] Deberardinis RJ, Lum JJ, Thompson CB (2006) Phosphatidylinositol 3-kinase-dependent modulation of carnitine palmitoyltransferase 1A expression regulates lipid metabolism during hematopoietic cell growth. J Biol Chem 281: 37372–37380. 10.1074/jbc.M608372200 17030509

[27] Kawano Y, Cohen DE (2013) Mechanisms of hepatic triglyceride accumulation in non-alcoholic fatty liver disease. J Gastroenterol 48: 434–441. 10.1007/s00535-013-0758-5 23397118 PMC3633701

[28] Fountas A, Diamantopoulos LN, Tsatsoulis A (2016) Erratum to 'Tyrosine Kinase Inhibitors and Diabetes: A Novel Treatment Paradigm?': [Trends in Endocrinology & Metabolism 26 (2015) 643–656]. Trends Endocrinol Metab 27: 65. 29496118 10.1016/j.tem.2015.11.006

[29] Mukai E, Ohta T, Kawamura H, Lee EY, Morita A, et al (2014) Enhanced vascular endothelial growth factor signaling in islets contributes to beta cell injury and consequential diabetes in spontaneously diabetic Torii rats. Diabetes Res Clin Pract 106: 303–311. 10.1016/j.diabres.2014.08.023 25262109

